# Factors Predicting Surgical Effort Using Explainable Artificial Intelligence in Advanced Stage Epithelial Ovarian Cancer

**DOI:** 10.3390/cancers14143447

**Published:** 2022-07-15

**Authors:** Alexandros Laios, Evangelos Kalampokis, Racheal Johnson, Sarika Munot, Amudha Thangavelu, Richard Hutson, Tim Broadhead, Georgios Theophilou, Chris Leach, David Nugent, Diederick De Jong

**Affiliations:** 1 Department of Gynaecologic Oncology, St James’s University Hospital, Leeds LS9 7TF, UK; racheal.johnson2@nhs.net (R.J.); s.munot@nhs.net (S.M.); amudhathangavelu@nhs.net (A.T.); richard.hutson@nhs.net (R.H.); tim.broadhead@nhs.net (T.B.); georgios.theophilou@nhs.net (G.T.); david.nugent@nhs.net (D.N.); diederick.dejong@nhs.net (D.D.J.); 2 Information Systems Lab, University of Macedonia, 54636 Thessaloniki, Greece; ekal@uom.edu.gr; 3 School of Human & Health Sciences, University of Huddersfield, Huddersfield HD1 3DH, UK; chris.leach1945@gmail.com; 4 Department of Psychology Services, South West Yorkshire Mental Health NHS Foundation Trust, The Laura Mitchell Health & Wellbeing Centre, Halifax HX1 1YR, UK

**Keywords:** Explainable Artificial Intelligence, complete cytoreduction, epithelial ovarian cancer, surgical complexity score, human factors

## Abstract

**Simple Summary:**

In the era of personalized medicine, Artificial Intelligence (AI) has emerged as a powerful tool with growing applications in the field of gynaecologic oncology. However, AI applications are encountered with several challenges derived from their “black-box” nature, which limits their adoption by clinicians. Surgical decision-making at cytoreductive surgery for epithelial ovarian cancer (EOC) is a complex matter, and an accurate prediction of surgical effort is required to ensure the good health and care of patients. We combined high-performance AI modeling with an eXplainable Artificial Intelligence (XAI) framework to explain feature effects and interactions associated with specific threshold surgical effort using data from a single public institution. We revealed features not routinely measured in the clinical practice, including human factors that could be responsible for the variation in the surgical effort. Selective decreased surgical effort may be associated with the surgeon’s age. The use of XAI frameworks can provide actionable information for surgeons to improve patient outcomes in gynaecologic oncology.

**Abstract:**

(1) Background: Surgical cytoreduction for epithelial ovarian cancer (EOC) is a complex procedure. Encompassed within the performance skills to achieve surgical precision, intra-operative surgical decision-making remains a core feature. The use of eXplainable Artificial Intelligence (XAI) could potentially interpret the influence of human factors on the surgical effort for the cytoreductive outcome in question; (2) Methods: The retrospective cohort study evaluated 560 consecutive EOC patients who underwent cytoreductive surgery between January 2014 and December 2019 in a single public institution. The eXtreme Gradient Boosting (XGBoost) and Deep Neural Network (DNN) algorithms were employed to develop the predictive model, including patient- and operation-specific features, and novel features reflecting human factors in surgical heuristics. The precision, recall, F1 score, and area under curve (AUC) were compared between both training algorithms. The SHapley Additive exPlanations (SHAP) framework was used to provide global and local explainability for the predictive model; (3) Results: A surgical complexity score (SCS) cut-off value of five was calculated using a Receiver Operator Characteristic (ROC) curve, above which the probability of incomplete cytoreduction was more likely (area under the curve [AUC] = 0.644; 95% confidence interval [CI] = 0.598–0.69; sensitivity and specificity 34.1%, 86.5%, respectively; *p* = 0.000). The XGBoost outperformed the DNN assessment for the prediction of the above threshold surgical effort outcome (AUC = 0.77; 95% [CI] 0.69–0.85; *p* < 0.05 vs. AUC 0.739; 95% [CI] 0.655–0.823; *p* < 0.95). We identified “turning points” that demonstrated a clear preference towards above the given cut-off level of surgical effort; in consultant surgeons with <12 years of experience, age <53 years old, who, when attempting primary cytoreductive surgery, recorded the presence of ascites, an Intraoperative Mapping of Ovarian Cancer score >4, and a Peritoneal Carcinomatosis Index >7, in a surgical environment with the optimization of infrastructural support. (4) Conclusions: Using XAI, we explain how intra-operative decisions may consider human factors during EOC cytoreduction alongside factual knowledge, to maximize the magnitude of the selected trade-off in effort. XAI techniques are critical for a better understanding of Artificial Intelligence frameworks, and to enhance their incorporation in medical applications.

## 1. Introduction

Cancer of the fallopian tube, ovary, or peritoneum (EOC) is the leading cause of death from gynaecological malignancy in the western world [1]. Over 70% of women diagnosed with EOC have advanced disease at presentation (FIGO stage 3–4) [1]. Surgical cytoreduction combined with platinum-based chemotherapy, either as treatment following surgery (adjuvant, ACT) or as treatment both before and after surgery (neoadjuvant, NACT) has long been the cornerstone in the treatment of advanced stage EOC [2,3]. The surgery aims at maximal cytoreduction for all visible disease, ideally reaching a total macroscopic tumor clearance. In EOC, the survival outcomes are inversely related to the initial tumor load and the residual disease (RD) following cytoreductive surgery [4]. Currently, the majority of surgeons performing cytoreductive surgery for EOC focus on efforts to achieve complete cytoreduction (R0 resection) as a primary outcome measure in both the primary debulking surgery (PDS) and the interval debulking surgery (IDS) setting [5].

Additional surgical procedures result in improved cytoreductive rates. This requires specialist training and surgical expertise, as well as a co-ordinated institutional effort to safely deliver [6]. In 2013, the National Institute for Health and Care Excellence (NICE) implemented guidelines for the widespread use of such procedures [7]. Nevertheless, the adaptation of the concept of (ultra-)radical surgery in advanced stage EOC, aiming at R0 resection, has been reportedly slow [8]. Based on the number and the complexity of the surgical procedures performed, the Aletti surgical complexity scoring system (SCS) was developed and validated to reflect the surgical effort [9]. In this approach, the extent of surgical complexity is determined by the total number of surgical procedures performed, rather than the intrinsic perceived risk of the individual procedures themselves.

In the realm of personalized medicine, innovative data mining technologies, such as Machine Learning (ML), a subfield of Artificial Intelligence, can be used for monitoring quality improvement and the effectiveness of the delivery of modern ovarian cancer care. The ML technology is exponentially growing, with potential applications being demonstrated across various domains of gynaecological oncology [10]. To mention but a few examples, ML can accurately classify benign and malignant ovarian neoplasms using the CEA and HE4 biomarkers, which assists with EOC early diagnosis and detection [11]. Based on multiple blood biomarkers, an EOC-specific predictive framework for clinical stage, histotypes, disease burden, and prognosis using ML has been introduced [12]. We previously investigated survival predictions in advanced stage EOC, using clinical variables. We applied ML-based feature selection to build models for two-year prognosis prediction, with satisfactory accuracy [13]. The end-point of such discoveries is their effective translation into patient care workflows.

The quality of surgical care is affected to a varying degree by decisions made by caregivers and patients throughout their management. Gynaecological Oncology surgeons are often required to make complex, difficult decisions at surgery. They must address modifiable risk factors, engage patients, balance risks, and optimize resources, in addition to the stress of conducting an operation. Judgment errors, such as decision making, are the second most common cause of preventable harm incurred to surgical patients [14]. The operation theater is a dynamic environment, where not only the lead surgeon, but also the anesthetists, assistant surgeons, circulators, and nurses play an important role in the success of the overall process. For example, due to time constraints, surgeons may often opt out of an analytical strategy to evaluate alternative courses of action. The quality of surgical decision-making is influenced by patient values and emotions, patient-surgeon interactions, resource availability, uncertainty, hypothetico-deductive reasoning, and individual judgment [15]. Patient values are individualized by nature, precluding the standardization of optimal decision-making [16]. Cancer surgery frequently evokes fear and anger, which influences the perceptions of risks and benefit [17]. There is a paucity of procedural data in relation to surgery, due to a multitude of regulatory, technical, and sociological factors.

Traditional clinical decision support tools, such as the National Surgical Quality Improvement Program (NSQIP) Surgical Risk Calculator [18], or the P-Possum score [19], can potentially reduce variability and mitigate risks. Such attempts to describe the post-operative course for individual patients have proven difficult in the gynaecological oncology setting. They appear to be of limited value in accurately predicting the risk of major complications following multi-visceral resections in EOC patients [20].

Intra-operative decision-making is a key element in the surgical management of EOC, yet it has received scant attention in the surgical literature. Historically, surgeons relied upon their experience to make consequential surgical decisions. There is an increasing need to inform decision-making during EOC cytoreduction by using predictive analytics. Machine Learning models, fed with fully curated patient- and tumor-specific data, could integrate features related to human factors to augment surgical decision-making. At the same time, explainability methods are required to render ML technologies more attractive to clinicians. Such methodology is still in its infancy, resulting in doubts regarding the usefulness of AI. Explainable Artificial Intelligence (XAI) techniques have only recently been introduced to explain the decisions made by ML models [21]. Their scope is to produce more explainable models whilst maintaining a high level of learning performance. We hypothesized that surgical decision-making during EOC cytoreduction is a complex process, thought to require real-time factual knowledge at the operation table, and what we might call declarative “hunches” (practical knowledge pertaining to previous surgical experience). We aimed to develop a data-driven procedural-based framework using modern ML to quantify the influence of features, including human factors in the tumor- and patient-specific crosstalk for predicting the account of surgical effort towards surgical cytoreduction. The study was designed to support the global and local explainability of the prediction problem (above the given threshold surgical effort) and the evaluation of interpretability by analyzing feature interactions using the prospectively registered data of EOC patients who received surgical treatment. The primary outcome was the construction and performance of the ML model to predict the account of surgical effort resulting in incomplete cytoreduction, and the development of XAI methodologies to explain the ML prediction by establishing and investigating the data-driven relationship between features.

## 2. Materials and Methods

Prospective registered data in the hospital-wide Patient Pathway Manager (PPM) database from 576 consecutive AOC women who underwent cytoreductive surgery with the intention to treat at St. James’s University Hospital, Leeds by a certified Gynaecological Oncology Surgeon from January 2014 to December 2019, was analyzed. This database was developed internally for clinical trials, and integrated with an electronic patient record system [22]. Our hospital is a tertiary center, recently accredited by the European Society of Gynaecological Oncology (ESGO) as a center of excellence for ovarian cancer surgery. The staging was reported by the 2014 International Federation of Gynaecology and Obstetrics (FIGO) classification [23].

Women underwent either PDS or 3–4 cycles of NACT followed by IDS if they had: FIGO stage 4 disease; poor performance status (PS); uncertainty regarding optimal tumor removal. Only advanced stage EOC patients with at least one pre-treatment CA125 value were included in the study. Excluded were patients aged <18 years, as well as those with non-epithelial histology, synchronous primary malignancy, and those undergoing secondary cytoreduction for recurrent disease. Patients who had progressive disease following three courses of NACT were excluded from analysis. Patients with low grade EOC were offered ACT, but were counseled regarding the chemo-resistant nature of the disease, and therefore, the limited lack of efficacy. The study was conducted according to the guidelines of the Declaration of Helsinki, and approved by the Leeds Teaching Hospitals Trust Institutional Review Board (MO20/133163/18.06.20), and informed written consent was obtained. All patients were discussed at the weekly central gynaecological oncology multidisciplinary team (MDT) meeting prior to treatment initiation.

All patients underwent the standard institutional therapy for ovarian cancer, namely primary surgical cytoreduction, which involved explorative laparotomy, abdominal hysterectomy with bilateral salpingo-oophorectomy omentectomy, and peritoneal sampling. This requires specialist training and surgical expertise, as well as co-ordinated institutional effort to safely deliver care [6]. Additional surgical procedures were performed according to practice recommendations from the British Gynaecological Cancer Society (BGCS), resulting in improved rates of cytoreduction [24]. Bulky lymph nodes were removed if this would complete macroscopic clearance. One colorectal ± upper abdominal surgeon with a specific interest in this area of joint working was available ad hoc for the provision of maximal effort cytoreductive surgery. The extent of support varied depending on the skills and the experience of the gynaecological oncology surgeon.

The enhanced Recovery after Surgery (ERAS) pathway, a multimodal peri-operative protocol designed to improve patient outcomes and speed recovery [25], was implemented at our center in late 2015. The Society guidelines for the peri-operative care of gynaecological oncology patients have been released and recently updated [26]. Post-operative critical care unit (CCU) admission was electively booked for high-risk EOC [27], who were scheduled to undergo complex major surgery, including multi-visceral resections, or undertook pre-operative cardiopulmonary exercise (CPEX) fitness testing if they were deemed high risk at pre-assessment.

Historically, survival outcomes for EOC in our center have been well above the UK average [28]. Most of the quality indicators formulated by the European Society of Gynaecological Oncology (ESGO) for ovarian cancer surgery in 2016 [29] had already been present. Nevertheless, a paradigm shift was initiated in the years 2016 and 2017 to facilitate performance of more complex multi-visceral surgery. This included strengthening the Gynaecological Oncology workforce by the appointment of three consultant colleagues; the development of governance models to support patient safety when undergoing maximal effort cytoreductive surgery for EOC by jointly working for gynaecological oncologists and surgeons from other disciplines; further and more robust optimization of the ERAS protocols with the appointment of specialized enhanced recovery nurses; facilitation of the logistical considerations to expand the availability of CCU admissions for high-risk EOC patients; intensification of their peri-operative management by dedicated anesthetists with a special interest in complex gynaecological oncology surgery, and meticulous reporting of the post-operative complications according to the Clavien-Dindo classification [30]. During this paradigm shift, the years 2016–2017 served as transition years for the full implementation of the ESGO benchmarks, which were further evaluated in the years 2018 and 2019.

Candidate predictors were selected a priori from three domains:

- Patient: age, year of diagnosis, year of surgery, Eastern Co-operative Oncology Group (ECOG) performance status (PS), histology type, grade (low and high), and stage (FIGO 3 or 4), pre-treatment, and pre-surgery Ca125.
- Operative/tumor factors: timing of surgery (PDS or IDS), presence of ascites (yes/no), intra-operative blood transfusion (yes/no), site of intra-operative bulk of the disease, size of the largest bulk of the disease, PCI and intra-operative mapping of ovarian cancer (IMO).
- Human factors addressing surgical heuristics: age of consultant surgeon, years of experience as a consultant, volume case within the cohort, and training status, i.e., whether the consultant was trained within the institution or not.

Patient and operation-specific variables are readily available in tertiary centers. They have been previously shown to be independent predictors of post-operative morbidity and mortality for ovarian cancer patients [3]. Several simple heuristic rules were considered for approximating human factors. These heuristics were motivated by sales and financial crisis forecasting, being a common and essential use of ML [31]. By setting a look-back period, such ML models can make standard non-dynamic predictions using actual sales from prior months; thus mimicking heuristics referring to previous clinical experience.

At the beginning of the surgery, the Peritoneal Carcinomatosis Index (PCI) was calculated to describe the extent of tumor load [32]. It appears to be precise and reproducible for the assessment of peritoneal dissemination. The intra-operative location of the disease was assessed using the IMO score at laparotomy [33]. The surgical complexity score (SCS) was used to describe the surgical effort. It was assigned based on the Aletti classification, but was examined as a continuous dependent variable [9]. Complete macroscopic cytoreduction was defined as macroscopic tumor clearance with no residual visible disease, as documented by a comprehensive visual assessment of all the areas of the abdomen.

Descriptive statistics were displayed using the frequencies and percentages for the binary and categorical variables, and using means and standard deviation (SD) or medians (with lower or upper quartiles for continuous variables). Chi-squared tests and Fisher exact tests were used for categorical variables and binary variables, respectively. Different cut-off points were tested using Receiver Operator Characteristic (ROC) curves to determine the most meaningful cut-off point. The statistical significance was set at *p* < 0.05. All analyses were performed with IBM SPSS Statistics for Windows (version 27.0).

To translate a continuous variable into a clinical decision, it is necessary to determine a cut-off point, classifying patients into two groups, each requiring a different type of intervention. One way to determine cut-off points is based on the distribution of the continuous variable, or through correlation with information on clinical or survival outcomes. For the binary prognosis classification, two groups were defined using the optimal cut-off from the ROC analysis. Those patients above the cut-off were placed in the positive group, whereas those below the cut-off were placed in the negative group.

Both a gradient boosting and a deep neural network (DNN) solution were used to create the predictive model. Although gradient boosting solutions are more suitable for tabular data, neural networks are occasionally able to capture signals better than gradient boosting models.

The dataset was initially split into training and test cohorts (70%:30% ratio) with repeated random sampling, until there was no significant difference (*p* = 0.20) between the two cohorts with respect to all variables. The training cohort was used to create and fine-tune the predictive model by selecting the set of features that maximize the model performance. To this end, five-fold stratified cross-validation (CV) was employed. Stratified folds were created to ensure the same distribution of negative and positive classes in each fold compared with the entire training dataset, when performing the CV evaluation.

First, the eXtreme Gradient Boosting (XGBoost), which is an implementation of a generalized gradient boosting algorithm, was employed [34]. Boosting refers to the general problem of boosting the performance of weak learning algorithms by combining all the generated hypotheses into a single hypothesis. The idea of boosting was further elaborated in gradient boosting, where one new weak learner is added at a time, and existing weak learners in the model are frozen and left unchanged. Specifically, XGBoost creates trees based on the previous tree’s residuals. New trees are created that predict the residuals or errors of prior trees, and then added together to make the final prediction. To maximize model performance, we investigated the combined effect of 20 parameters by evaluating a grid of 8000 combinations of values using the Scikit-learn’s GridSearchCV function. The scale_pos_weight hyperparameter of XGBoost tuned the behavior of the algorithm for imbalanced classification problems. The scale_pos_weight value was used to scale the gradient for the positive class, and thus to achieve better performance when making predictions on the positive class. The value of this hyperparameter was set to the imbalance ratio of the response variable in our dataset.

Regarding the DNN, we employed TensorFlow, with its high-level API, Keras, integrated with more classical approaches based on Scikit-learn and Pandas. Layers in the deep learning model can define the model architecture. We employed dense layers; i.e., the neurons of a layer were connected to every neuron of its preceding layer. Results from each neuron from the preceding layers traverse towards every single neuron of the dense layer. In order to create the final DNN solution, different values for the main characteristics and hyperparameters were considered. First, although ReLU is the most popular activation function, a series of non-monotonic activation functions, such as GeLU, SeLU, and MiSH, were recently proposed. The activation function is a function that is used for the transformation of the input values of neurons. According to the literature, GeLU, SeLU, and MiSH are more suitable for modeling tabular data [35]. The batch size defines the number of samples that will be propagated through the network. A training dataset can be divided into one or more batches. The number of epochs is a hyperparameter that defines the number of times that the learning algorithm will work through the entire training dataset.

To evaluate the effectiveness of the models, we considered multiple metrics that could also capture the balance of the data classes, irrespective of the prediction accuracy. Performance metrics included Area Under Curve (AUC) of both the Receiver Operating Characteristic (ROC) and the Precision-Recall curves, as well as precision, recall, and f1 score, on both minority and majority classes.

An explanation is the collection of features in the interpretable domain that contributed to the classification problem. To explain the predictive models, we proposed the Shapley Additive Explanations (SHAP) values as a unified measure of feature importance [36]. This is a game theory-inspired method that attempts to enhance interpretability by computing the importance values for each feature for individual predictions. The method explains a model globally by expressing it as a linear function of features. In other words, it explains how much the presence of a feature contributes to the model’s overall predictions.

## 3. Results

A total of 560 EOC patients with histological confirmation were enrolled in the study (Table 1). A small cohort of consultant surgeons (*n* = 8) performed cytoreductive surgery during the study period. The mean age of patients in the entire cohort was 64 ± 11 years, with the SCS < 5 group being reliably older than the SCS > 4 group (*p* = 0.003). In addition, ECOG PFS at diagnosis, the timing of surgery, pre-treatment CA125, and also real-time metrics such as the size of the largest tumor deposit, PCI, IMO, and intra-operative ascites also differed reliably between the two groups. With respect to the human factors, consultant age, years of consultant experience, and training status but not caseload, were distributed differently between the two groups.

**Figure 1:**
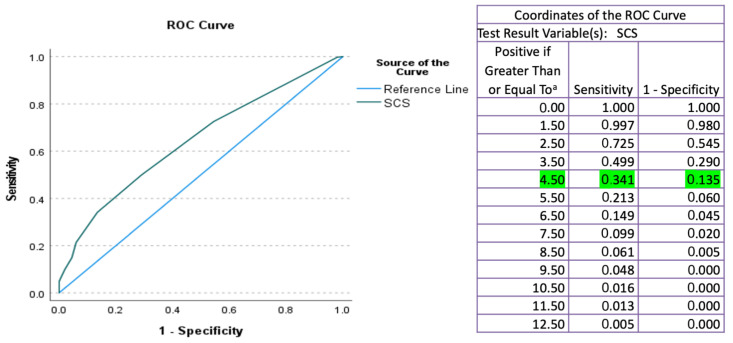
Receiver Operator Characteristic (ROC) curve for Surgical Complexity Score (SCS) to detect the cut-off value that predicts incomplete cytoreduction. A cut-off value of 4.5 was calculated, above which R0 resection is not achievable with a specificity of 86.5% (AUC = 0.644 CI = 0.598–0.69).

To predict that R0 resection is not achievable, the Specificity needs to be high, irrespective of Sensitivity (as in a screening test imitated during pre-operative patient counseling). An ROC curve plotted the SCS score to detect the optimal cut-off value to predict non-RO gave a sufficiently high specificity of 86.5% using a cut-off of 4.5, above which incomplete cytoreduction is expected (AUC = 0.644; 95% Confidence Interval [CI] 0.598–0.690; sensitivity and specificity 34.1%, 86.5%; respectively; *p* = 0.00) (Figure 1). The XGBoost accurately predicted (AUC = 0.77; 95% [CI] 0.69–0.85; *p* < 0.05).

The performance results of the created model are shown in Table 2.

We created a sequential DNN model with two dense layers of 200 nodes. The DNN accurately predicted the outcome (AUC 0.739; 95% [CI] 0.655–0.823; *p* < 0.95), but underperformed compared with the XGBoost model (Table 3).

To promote reproducibility, both model hyperparameters can be found in Table 4.

### 3.1. Feature Analysis

To demonstrate the value of our model’s explained predictions and to refine features influencing incomplete cytoreduction, we developed: (a) SHAP Summary plots for global and local explanations of the results; (b) SHAP Dependence plots of the key risk features for SCS > 4; (c) SHAP interaction Value Dependence plots; and (d) SHAP Decision plots that explain the SC > 4 risk prediction for individual patients.

#### SHAP Summary Plots

**Figure 2:**
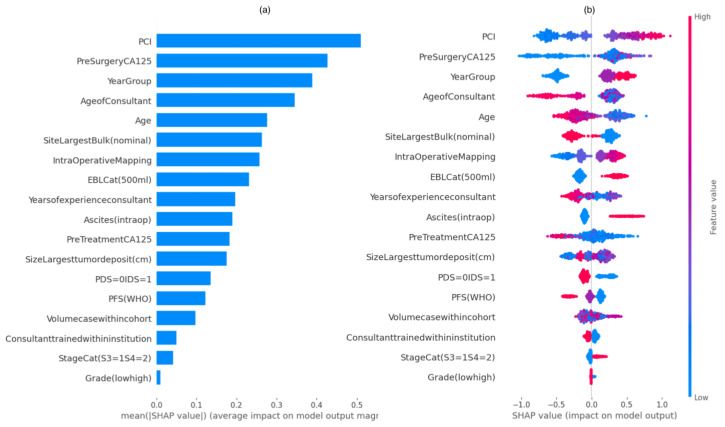
(**a**) Feature importance bar plot description of SHAP values (left) and (**b**) summary plot showing a set of beeswarm plots of feature distribution for global explainability of threshold SCS prediction (right). Dots correspond to the individual EOC patients. SCS, surgical complexity score; EOC, epithelial ovarian cancer.

The SHAP Summary plot was presented in the form of a set of beeswarm plots (Figure 2). The order of the features reflected their importance; i.e., the sum of the SHAP value magnitudes across all the samples. Each point on the summary plot is a Shapley value for a feature and an instance. The position on the *y*-axis is determined by the feature, and on the *x*-axis by the Shapley value. The color represents the value of the feature, from blue = low to red = high. The PCI was the most important feature, globally. The plot indicates the direction of the effects, so, for example, a lower PCI had a higher probability for decreased surgical effort (SCS < 5), translating into a lower probability for achieving R0 resection than with a higher PCI. Similarly, lower SHAP values of pre-surgery CA125 values and IMO scores, and earlier years at surgery corresponded to a lower probability for increased surgical effort (SCS > 4). The top-five list of important features was completed by patient age and age of consultant at surgery, with older patients and consultants associated with a lower surgical effort than younger patients and consultants. The plot also presents the distribution of effect sizes, such as the long tails of several features. These long tails suggest that features with low global importance can be equally important for specific data zones. For example, pre-treatment CA125 value and volume case within the cohort are not high up in the list of important features; however, in several cases, they may have an effect on surgical effort.

### 3.2. SHAP Dependence Plots of Human Intuition Features

**Figure 3:**
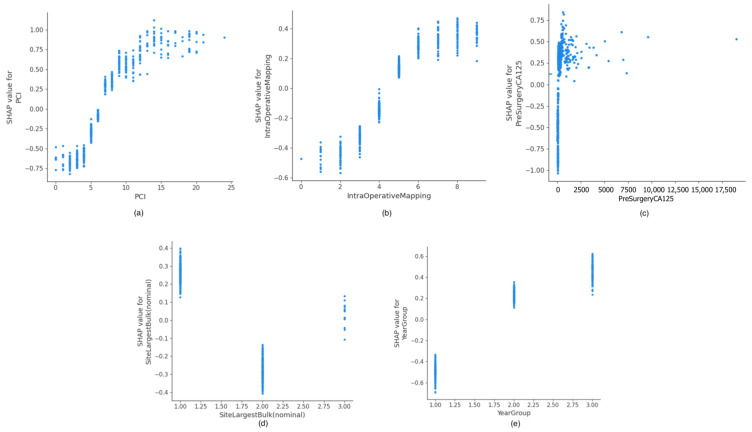
Examples of SHAP Value Dependence plots for top global explainability features showing the impact of each feature value on the prediction: (**a**) PCI, (**b**) Intra-Operative Mapping, (**c**) Pre-Surgery CA125, (**d**) Size of Largest Tumor, (**e**) Year of Surgery. PCI, Peritoneal Carcinomatosis Index.

**Figure 4:**
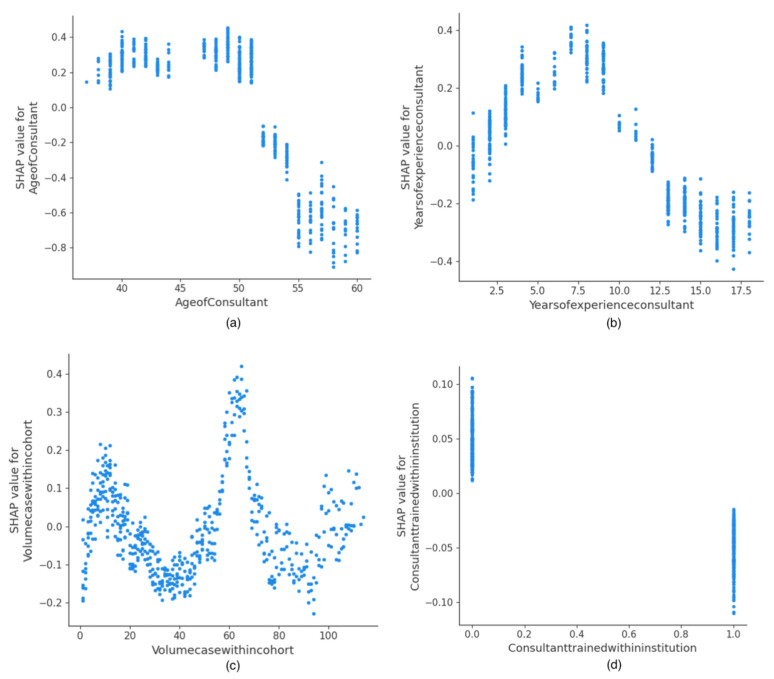
Examples of SHAP Value Dependence plots for global explainability features reflecting human factors showing the impact of each feature value on the prediction: (**a**) Consultant Age, (**b**) Years of Experience, (**c**) Volume Case within Cohort, (**d**) Site of Consultant Training.

The SHAP dependence plot reveals the impact of each feature’s value on the prediction problem (Figure 3 and Figure 4). It plots the value of a feature on the x-axis, and the SHAP feature value on the y-axis by changing a specific feature in the model each time. Figure 3a,b clearly shows the inflection points of the PCI and IMO scores on the surgical effort. For PCI < 8 and IMO < 5, the overall SHAP values are negative, and a lower surgical effort (SCS < 5) is likely to be exerted. If pre-surgery CA125 is not within normal range, the overall SHAP values are positive and a higher surgical effort (SCS > 4) is likely to be exerted (Figure 3c). The overall SHAP values are positive for size of largest deposit <5 cm, but are negative for size of largest deposit 3–10 cm (Figure 3d). Earlier (baseline) years at surgery show lower surgical effort than transition and evaluation years (Figure 3e). With respect to human factors, the impact of consultant age on the surgical effort is shown in Figure 4a. For consultant age <53 years old, the overall SHAP values are positive, and a higher surgical effort (SCS > 4) is likely to be exerted. Then, the SHAP values become negative, which means that by increasing consultant age, the probability for less surgical effort (SCS < 5) increases. A similar trend is observed if years of consultant experience is selected as a feature to determine its impact; >12 years of consultant experience is associated with a probability for lower surgical effort (Figure 4b). Similarly, an inflection point within a range of 50–70 cases exhibits the sole pattern of higher surgical effort probability (Figure 4c). For those consultants trained within the institution, there was a clear trend towards more surgical cynicism (Figure 4d).

### 3.3. SHAP Value Interaction Plots of Features Related to Human Factors

**Figure 5:**
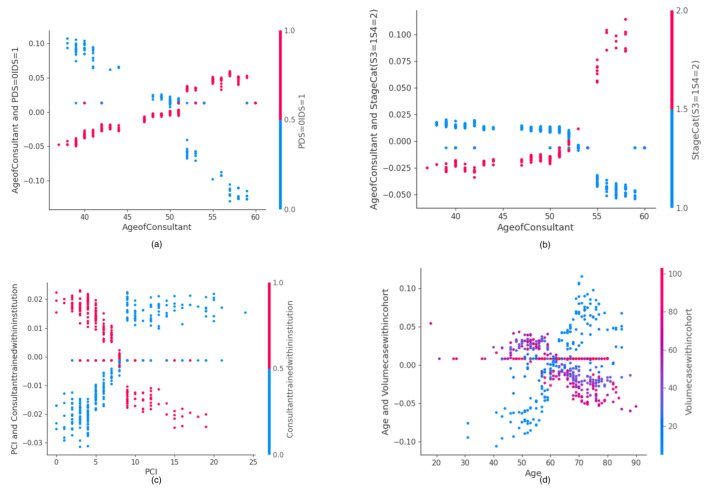
Examples of SHAP Value Interaction plots related to human factors: (**a**) Consultant age and timing of surgery, (**b**) Consultant age and stage, (**c**) PCI and site of consultant training, (**d**) Patient age and volume case within cohort. PCI, Peritoneal Carcinomatosis Index.

**Figure 6:**
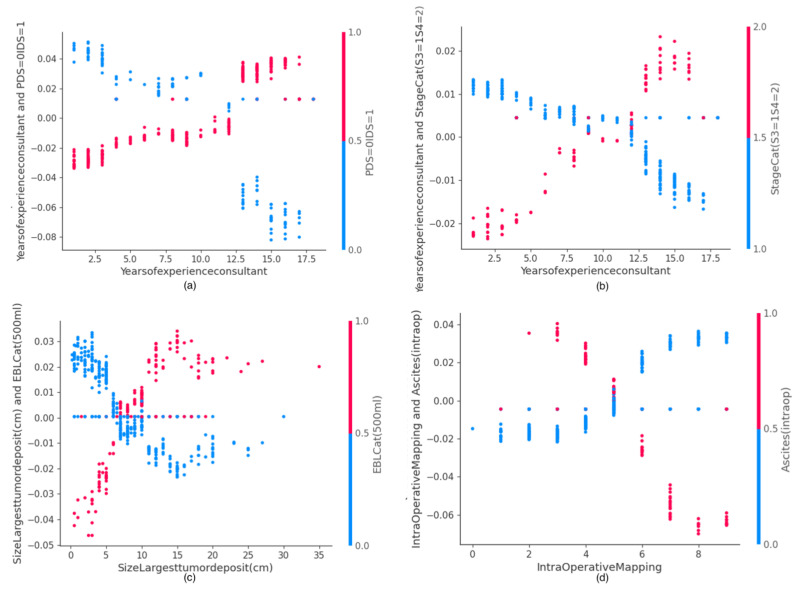
Examples of SHAP Value Interaction plots related to human factors: (**a**) Years of consultant experience and timing of surgery, (**b**) Years of consultant experience and stage, (**c**) Size of largest tumor and EBL, (**d**) Intra-Operative Mapping Score and ascites. EBL, Estimated Blood Loss.

The SHAP interaction values can be interpreted as the difference between the SHAP values for feature A when feature B is present, and the SHAP values for feature B when feature A is absent. An interaction feature is the additional combined feature effect after accounting for the individual feature effects. Examples of the plots of the SHAP interaction values of various pairs of features are shown for human factor features in Figure 5 and Figure 6.

The plots of the SHAP interaction value of consultant age with timing of surgery (Figure 5a) and cancer stage (Figure 5b) show how the effect of timing of surgery and cancer stage vary on the probability of surgical effort with the consultant age. Increased consultant age favors increased surgical effort for IDS and stage 4 disease, irrespective of patient age. Consultants trained within the institution are likely to exert higher surgical effort for PCI > 7 (Figure 5c). Consultants with a case volume >50, who are essentially more experienced, are likely to exert more surgical effort in patients >60 years old (Figure 5d). The plots of the SHAP interaction values of years of consultant experience with time of the procedure (Figure 6a) and disease stage (Figure 6b) show that years of consultant experience may have a different effect on the probability of surgical effort, depending on the timing of surgery and disease stage. Less surgical effort for PDS, compared with greater surgical effort for IDS, are likely to be exerted by surgeons with a consultant experience >12 years. Meaningful interactions were observed between size of tumor deposit and EBL (Figure 6c) or IMO and intra-operative ascites, consistent with prior knowledge (Figure 6d).

### 3.4. SHAP Decision Plots

**Figure 7:**
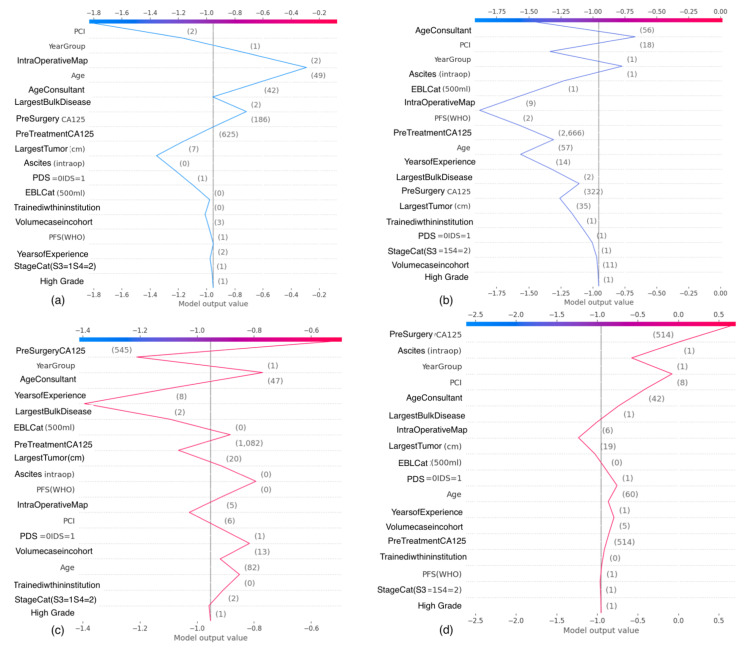
Examples of Decision plots based on feature integration by the XAI model into a single risk for prediction of surgical effort. For the probability of SCS > 4, blue features have values that increased the probability, while red features decreased the probability. The combination of impacts of all features is the predicted prediction risk for above threshold surgical effort. (**a**,**b**) The odds for SCS > 4 range between 1.40- and 1.80-fold higher than normal. (**c**,**d**) the odds for SCS < 5—which means that inomplete cytoreduction is most likely to happen—range between 1.53- and 1.8-fold higher than normal. Each feature impact value represents the change in risk when that feature’s value is known versus unknown. The examples clearly demonstrate the complex interactions between patients, surgeons, and ovarian cancer-specific features. SCS, Surgical Complexity Score.

For local explainability, the SHAP Force plots demonstrate features, each contributing to push from the base value (the average model output over the training dataset) to the model output value. Several examples referring to individual cases are illustrated (Figure 7). These literal representations of the SHAP values are akin to statistical linear models, where the sum of effects, plus the intercept, equals the prediction.

## 4. Discussion

**Figure 8:**
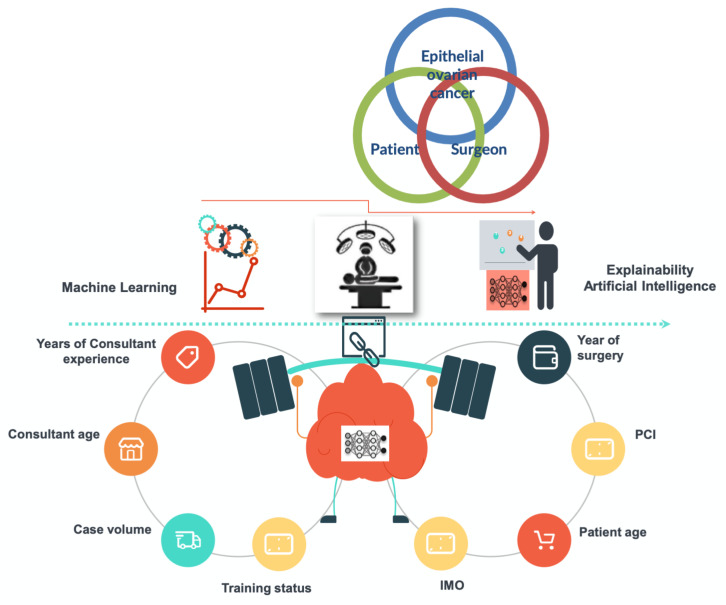
Schematic representation of our concept, exploring features affecting surgical effort at cytoreductive surgery for EOC. Explainable AI is employed to interpret the fine balance between factual knowledge and surgical heuristics.

It is becoming imperative to apply AI models that are inherently interpretable, together with XAI methods to tally accurate predictions with a better understanding of the underlying reasoning of the AI approaches. The need for using XAI techniques to explain individual decisions of predictive models has only recently emerged [37]. The use of XAI in oncology is still in its infancy, and is not widely appreciated. This study is the first to our knowledge, to attempt to implement XAI models to explain the prediction of surgical effort at EOC cytoreduction, by feeding the models with features that also include human factors. Our team has pioneered the implementation of XAI models in the field of gynecological oncology [38]. In our previous work, we demonstrated the ability of XAI to predict the outcomes of surgical cytoreduction. In addition, we identified non-linear interactions between decreased R0 resection rates and increased PCI scores. In this work, we showcased how to preserve the model prediction accuracy and to retain interpretability by developing a methodology to provide justified explanations of the value of the surgical effort when RO resection is not practically achievable. We produced powerful visualizations to illustrate that surgical effort shows significant variation based on reasoning from pre- and intra-operative information. Procedural data were holistically analyzed, together with features not routinely examined, such as human factors (consultant age, years of experience, case volume within cohort, and training status) that were calculated in a possible/pragmatic manner, taking into account the resources available at the time (Figure 8). The XAI-based revelation of the critical inflection points enabled some fundamental understanding of the surgical decision-making for EOC, its variability, the crucial parameters, dependencies, and potentially hidden structures, thus addressing surgical heuristics. Equally, the cohort infers that surgical practice could change during this study period, as surgeons moved through their exponential practice from one age group to the other. The concept can also serve for context-aware assistance, because by knowing what surgical effort to exert at the beginning of surgery, one can predict the duration of the procedure, to facilitate scheduling, or to anticipate needs for resources. This would exemplify the integration of human factors into surgical care pathways. That being said, the data were not analyzed to reflect individual practice.

The predictive model was constructed based on the SHAP values of 18 features. This adds weight to the wide adoption of SHAP as an interpretability tool, as currently, there is no agreed XAI framework in the broad AI field. The top-five list of important features included pre- and intra-operative features, in addition to the presence of a dynamic surgical environment. Our results show that optimization of infrastructural support, a potentially modifiable, hence clinically meaningful factor, can help to extend the maximal effort approach in EOC patients, in line with other studies [39]. The most important finding is that the variability in surgical effort may be attributable to human factors. Indeed, consultant age was the most impactful human factor. Mechanisms to explain our exploratory results could be attributed to the “age effect” (consultant age and years of experience), rather than the “cohort effect” (training status and case volume). However, the appointment of new consultant surgeons in the transition years of our study can be considered a “cohort effect” that has positively impacted on the adoption of increased surgical effort. We identified “turning points” in a surgeon’s career, with less surgical effort being exerted at >12 years of experience, and at >52 years of age. Ageing might be associated with reduced cognitive or motor function; however, age also relates to experience. A 10-year increase in surgeon age is associated with 5% relative decreased odds of a composite outcome that includes death, hospital re-admission, and post-operative complications [40]. How consultant age affects surgical effort is speculative, but interestingly, in our study, later-career surgeons showed more surgical scepticism for PDS and stage 4 disease. This interaction might be bi-directional, as surgeons might self-select or treat fewer patients over time, but our study did not confirm this. To a lesser extent, surgeons with a high case load, essentially more experienced ones, demonstrated greater surgical effort for the patients >60 years old. The volume of work is the basis for the evolution of a younger surgeon; so is surgical culture, mentoring by a senior surgeon, adherence to hospital guidelines, and litigation. Intra-operatively, the decision-making process occurs irrespective of the surgical skills. It takes approximately five years for a new surgeon to adjust to care according to nuances at all stages [41]. The older surgeon may “read the game”, and will intuitively understand when to play the winners, possibly from a recollection of similar scenarios; an extrapolation of its sport analogy [42]. That said, adopting a culture of surgical radicality would entail teaming early- and late-career surgeons to improve both of their experiences; the hallmark of the influential theory of group decision-making, and the so-called functional perspective [43]. Nevertheless, surgeon age is not an important predictor of operative risk [44].

Factual knowledge, expressed by PCI and IMO scores, had a positive influence on the surgical effort. This is in line with other studies showing that PCI is a strong predictor of incomplete cytoreduction in EOC [45]. In that same study, a cut-off of 24 or higher was related to incomplete cytoreduction, whilst in our study, increased surgical effort was more likely for a PCI > 7. The timing of surgery differed significantly between the two surgical cultures, but it remains not a significant factor in overall survival [46]. The positive correlation between IMO and SCS has been demonstrated by London research groups [47]. Notably, in our study, incorporating maximal effort surgery in the management of EOC patients did not come at the cost of delaying adjuvant chemotherapy, an important prognostic factor. Hence, the overall survivals between the below- and above-threshold SCS groups were not statistically significant (data not shown).

The local explainability of the model showed that factors globally determining the probability of increased or decreased surgical effort may, in extreme cases, be less important for individual patients (Figure 7). An example was given, where the presence of ascites and high pre-treatment CA125, despite an ECOG PS 2, prompted an older consultant to exert maximal surgical effort, having identified very widespread disease. As a result, XAI techniques can be used by care takers to make meaningful decisions that are specific to their patients. Gynaecological oncologists are frequently required to work together with other surgical disciplines to maximize surgical effort. In our institution, they often consult colorectal or hepatobiliary surgeons to reach a consensus for intra-operative decision-making, when required. Similar pathways have been adopted by many centers of excellence. Interaction is an advantage in group decision-making, reducing over-confidence bias and error rates [48]. This approach allows for patients with seemingly unresectable disease to receive and benefit from PDS when appropriate [49]. Nevertheless, as teams make decisions that are systematically different to lone individuals [50], our results may be potentially diluted.

Despite the careful selection of human factors, the study does not shed light into how intuitive judgments are formed by the operation table. Past attempts to articulate how surgical decisions are made in practice have underestimated the complexity of the process. During EOC surgery, we are often time-restricted from engaging into reasoning. Conversely, reasoning may need to be aborted when we run out of time, due to time constraints with theater lists. An evidence-based decision-making process is secured by domain expertise, and a minimum of five years additional practicing experience [41]. We theorize that younger surgeons may be more enthusiastic when exerting surgical effort, but there is a trend towards changing their practice if they had experienced poorer outcomes. A “hunch” approach may not be uncommon. After all, EOC surgical cytoreduction is “intuitive”, and has been introduced on the basis of non-randomized data; the evidence to support its use largely being in the form of retrospective data collections. What process do more experienced surgeons go through that allows them to decide to use less effort with selected patients? It is suggested that clinicians who are further from training are less likely to comply with new treatments, and they might rely more often on clinical evidence that is not up to date [51]. The concept of “previous clinical experience”, or the practical knowledge, in which some earlier cases were not successful, was demonstrated in our previous work, which employed the k-NN approach to accurately predict complete cytoreduction in advanced stage EOC surgery [52]. This might also suggest why some surgeons may become overwhelmed with the idea of case complexity and longer operations. Future research, including detailed interviews with surgeons, is expected to capture how all these factors affect the quality of decision making.

Evidence from the SCOTROC-1 trial study suggests that UK, compared to non-UK ovarian cancer patients, receive less extensive surgery, and are less likely to undergo complete cytoreduction [53]. At a time when surgeons are judged on the basis of markers of quality or the effectiveness of short-term care, it is not surprising that surgeons manifest considerable reluctance to operate on higher-risk patients with extensive tumor dissemination, thus requiring more complex surgery [49]. Equally, surgeons are often subjected to scrutiny as to why they consider extending their therapeutic effort to this challenging patient cohort with a presumed less favorable prognosis. As long as national targets and directives are given priority over patient care, a more defensive and risk-averse practice may prevail [54].

A recent anonymized UK survey confirmed, in line with our study, a variation in the surgical management of EOC amongst consultant gynecological oncologists. Based on the mean operating times, caseload, and types of procedures undertaken, the survey provided compelling evidence that in many UK cancer centers, the surgical goal has not been complete cytoreduction [8]. Departments and Regulatory Bodies need to estimate the probability of different outcomes following cytoreductive surgery. Without insightful data to reflect the actual practice, a disparity in outcomes is almost inevitable. The recently launched, jointly funded Ovarian Cancer Audit Feasibility Pilot in England prepares the ground for a crucial full-scale clinical audit to map ovarian cancer care, surgery, and survival across the NHS. We have continuously audited and recently published our surgical outcomes. We demonstrated that improving complete surgical cytoreduction rates in advanced stage EOC is achievable without a significant increase in morbidity [52]. Our complete surgical cytoreduction rates are comparable to other high-volume specialized centers [55]. The increased SCS demonstrated our more aggressive approach in cytoreductive surgery for EOC. Other methods, such as mindfulness practice among multidisciplinary teams, can improve communication, connectivity, and can be nuanced towards a better understanding of the safety culture [56]. The standardization of surgical practice and the identification of centers of excellence will potentially benefit patients from a maximal-effort approach at all possible levels [57].

The development of the ML predictive model was designed around the SCS. The SCS cut-off value was similar to the median SCS value. We did not use survival as an outcome, as we focused only on those EOC patients who received surgery. We acknowledge that this may potentially introduce selection bias when evaluating survival, as the denominator should include all presenting EOC patients; a factor that already has been emphasized as being essential for benchmarking and quality assurance elsewhere [58]. Alternative outcomes, such as 30-day readmission, were not considered, due to ongoing clinical concern in the primary treatment of EOC [59]. Equally, trade-offs between outcomes are inevitable. Better strategies for communicating surgical risks and benefits, several of which are already in development, will also help patients understand the complexities of the relevant trade-offs. In that respect, the SCS indicates the overall complexity of the procedure; albeit, certain surgical procedures do not translate into it. It is speculated that the expected postoperative morbidity may often be the only externally validated index for addressing the surgical complexity of EOC [9].

The study’s strength was the implementation of combined ML modeling with XAI that led to the development of the prediction algorithm for use in clinical decision-making from a binary classification framework that contained balanced data. While the cancer diagnostic process follows a regular flow of data acquisition, the surgical process varies significantly from case to case, and is highly specific to the procedure, the patient, and the surgeon [60]. Our model was populated by fully curated data from 560 EOC patients derived from a resourceful ovarian cancer database, which allowed for a standardized annotation of interventional care data and unconventional data representing human factors. We proved that XAI can also aid in the development of the predictive modeling, eliminating irrelevant features, leading to a model that is less prone to overfitting. The SHAP framework was used to identify the most important features to be included in the model. Indeed, we observed a significant overlap between the top five per sample (local) and per cohort (global) weighted features. Therefore, the main strength of this study is the two-level explainability; the per sample explainability of our results, including features not routinely examined in the clinical setting. The model performance was reliable and robust, which is important when decision-making refers to anatomical manipulations at surgery, which are frequently irreversible. Any preference for less accurate models, whether AI or human decision-making, carries obvious risks to patient health and beneficience. It was not surprising that the XGBoost performed better that the DNN algorithm for the prediction problem. The use of Deep Learning in not new in Gynaecological Oncology [61], with a selective preference for Gynaecological imaging, exhibiting a good diagnostic performance. The model appeared to be more precise for group 1 (SCS < 5); hence, it could potentially identify those patient groups, who, under the influence of human factors, might benefit from a less aggressive surgery to recover more rapidly, and then embark on timely adjuvant treatment.

Lastly, we presented a new class of plots, known as decision plots, to visualize the model’s predictions at the individual level, via the SHAP package. The magnitude and the direction of the major feature effects were made easy to identify. In this way, we clearly demonstrated a large number of feature effects, as well as the cumulative effect of the complex interactions between patients, surgeons, and ovarian cancer-specific features. Because the local explanation of the key features is not independent of the underlying AI model’s performance, where the certainty of predictions was not satisfactory, the explanation for several individuals could be irrational, which would make it more difficult to appreciate the relevant importance of the human factors.

The study comprised a single-institution experience, which may limit the generalizability of the conclusions. Hence, the applicability of these findings to other centers remains to be determined. Some aspects of data annotation are resource-intensive and require expertise for their content. A limitation of this study is the lack of prospective quality-of-life data. While the results of the TRUST trial are eagerly anticipated [62], the preliminary results from the SOCQER-2 study showed no association between surgical complexity and global health status at 12 months [63]. Moreover, extensive surgery did not seem to cause a decrease in patient quality of life, compared with the pre-operative scores.

The study was of a retrospective nature; not necessarily a limiting factor, being that human decisions are often interpretable and rationalized after the event. A lack of randomization to account for tumor burden at diagnosis could potentially bias the results, as patients receiving NACT might have had extensive disease, affecting the surgical effort during timely cytoreduction. We acknowledge that surgical practice might have evolved during the study period, but no individual or categorical data were sufficient to support practice change. In the small cohort of surgeons who performed EOC surgery, those outliers who did not cross both SCS threshold groups might have skewed the results. Ultimately, we have to admit the use of XAI—a likely game changer—is not without limitations, due to the inherent inaccuracies when attempting to explain the AI models; explanations can be often approximations or probabilistic estimates, because reproducing reality can be computationally prohibitive [64]. Surgical decision making is also heavily regulated, because of its MDT nature [65]; hence, there is perhaps little space for substantial contribution by human factors, derived from the rather interdisciplinary nature of the AI tool. Clinical validation on heterogenous data sources is required to reduce the risk of potential AI bias. Although our findings should be considered with caution, the study reveals the enormous potential of the ML tools exerting their effects on surgical decision-making. Our results would have been confounded, should another outcome or threshold level be selected, unmeasured by our analysis. This state of affairs can be potentially addressed by the means of “value-fexible” AI that provides different options for the patient [66]. One treatment rarely, if ever, is definitively shown to improve outcomes for all important end points [67]. Given the clear potential, future integration in the clinical practice will warrant the protocol standardization of feature extraction, and prospective external validation on large cohorts.

## 5. Conclusions

Surgical decision-making at EOC cytoreduction, a complex procedure, is critically layered upon situational awareness and the impact of human factors (Figure 8). We employed the SHAP framework to provide per sample (local) and per cohort (global) feature set explainability for the decisions made by the high-performance XGBoost ensemble tool, which outperformed the DNN assessment tool, and was prioritized over its predictive accuracy. We demonstrated a fine balance between predictive accuracy and descriptive interpretability, and we pinpointed the most salient feature interactions. Thanks to the innovative XAI approach, we learnt how augmenting machine explanations with self-interpretable data could enhance perceived control over the decision-making process and clinical acceptance. Selected decreased surgical effort may be associated with surgeon age. Future research will investigate the processes behind surgical decision-making and the role of surgical heuristics. The use of XAI frameworks can provide actionable information for surgeons to improve patient outcomes in gynaecologic oncology.

## Figures and Tables

**Table 1:** A Two Independent Sample *t* Test was used for continuous variables, whereas a Chi-Squared Test of Independence was used for categorical data. Statistical analysis was performed using Python’s SciPy library. Values are mean ± SD or *n* (%).

Clinical Characteristics ^1^	Overall (*n* = 560)	Train Set (*n* = 392)	Test Set (*n* = 168)	*p*-Value	SCS > 4 Group (*n* = 165)	SCS < 5 Group (*n* = 395)	*p*-Value
Age	64 ± 11	64 ± 11	63 ± 11	0.76	61 ± 12	64 ± 11	0.003
High grade	504 (90%)	354 (90%)	150 (89%)	0.83	144 (87%)	360 (91%)	0.21
Stage 3	406 (72%)	287 (73%)	119 (71%)	0.63	113 (68%)	293 (74%)	0.2
PFS (WHO) at diagnosis				0.69			0.001
0	266 (47%)	183 (47%)	83 (49%)		95 (58%)	171 (43%)	
1	208 (37%)	151 (38%)	57 (34%)		55 (33%)	153 (39%)	
2	67 (12%)	47 (12%)	20 (12%)		8 (5%)	59 (15%)	
3	17 (3%)	10 (2%)	7 (4%)		5 (3%)	12 (3%)	
4	2 (0.3%)	1 (0.2%)	1 (0.5%)		2 (1%)	0 (0%)	
Age of Consultant	49 ± 6	49 ± 6	49 ± 6	0.31	48 ± 6	50 ± 6	0.001
Volume case within cohort	45 ± 31	46 ± 32	44 ± 29	0.36	46 ± 30	45 ± 32	0.83
Years of experience	10 ± 5	10 ± 5	9 ± 5	0.34	9 ± 5	10 ± 5	0.001
Consultant trained within the institution	250 (45%)	181 (46%)	69 (41%)	0.3	60 (36%)	190 (48%)	0.01
Timing of surgery							0.001
Interval Debulking	388 (69%)	274 (70%)	114 (68%)	0.7	90 (55%)	298 (75%)	
Primary Debulking	172 (31%)	118 (30%)	54 (32%)		75 (45%)	97 (25%)	
Year				0.96			0.001
Baseline 2014-15	184 (33%)	128 (33%)	56 (33%)		22 (13%)	162 (41%)	
Transition 2016-17	195 (35%)	136 (35%)	59 (35%)		67 (41%)	128 (32%)	
Evaluation 2018-19	181 (32%)	128 (33%)	53 (31%)		76 (46%)	105 (26%)	
EBL > 500 mL	179 (32%)	128 (33%)	51 (30%)	0.66	86 (52%)	93 (24%)	0.001
Pre-Treatment CA125	1515 ± 2710	1545 ± 2762	1445 ± 2592	0.68	1237 ± 2318	1630 ± 2853	0.088
Pre-Surgery CA125	411 ± 1175	381 ± 899	476 ± 1649	0.91	443 ± 968	397 ± 1252	0.64
Size Largest Tumor Deposit (cm)	8.9 ± 5.6	9 ± 5.4	8.5 ± 6	0.34	10.4 ± 5.5	8.3 ± 5.5	<0.001
PCI	7 ± 4	7 ± 4	7 ± 5	0.78	10 ± 5	6 ± 4	0.001
Largest Bulk of disease (nominal)				0.88			0.99
Ovary	294 (52%)	207 (53%)	87 (52%)		92 (56%)	202 (51%)	
Omentum	252 (45%)	176 (45%)	76 (45%)		66 (40%)	186 (47%)	
Miscellaneous	14 (2%)	9 (2%)	5 (3%)		7 (4%)	7 (2%)	
Intra Operative Mapping	5 #xB1; 2	5 ± 2	5 ± 2	0.62	6 ± 2	5 ± 2	0.001
Ascites (intra-op) (mL)	130 (23%)	93 (24%)	37 (22%)	0.74	57 (35%)	73 (18%)	0.001

^1^ PFS; performance status, EBL; estimated blood loss, PCI; peritoneal carcinomatosis index, SCS; surgical complexity score.

**Table 2:** XGBoost: precision, recall, and F1-score for both classes, namely Group 1 (SCS < 5) and Group 2 (SCS > 4).

	Precision	Recall	F1-Score
Group 1 (SCS < 5)	0.84	0.77	0.80
Group 2 (SCS > 4)	0.56	0.67	0.61

**Table 3:** DNN: precision, recall, and F1-score for both classes, namely Group 1 (SCS < 5) and Group 2 (SCS > 4).

	Precision	Recall	F1-Score
Group 1 (SCS < 5)	0.84	0.69	0.76
Group 2 (SCS > 4)	0.51	0.71	0.59

**Table 4:** Hyperparameters for both models, namely XGBoost and DNN.

Algorithm	Hyperparameters ^1^
XGBoost	‘max_depth’: 3, ‘alpha’: 0.001, ‘subsample’: 0.75, ‘learning_rate’: 0.01, ‘n_estimators’: 500, ‘colsample_bytree’: 0.75, ‘colsample_bylevel’: 0.75, ‘scale_pos_weight’: 2.39
DNN	activation function: GeLU, dense dropout: 0.1, learning rate: 0.01, batch size: 20, epochs: 9

^1^ XGBoost hyperparameters: ‘max_depth’, maximum depth of a tree; ‘alpha’, L1 regularization term; ‘subsample’, subsample ratio of the training instances; ‘learning_rate’, step size shrinkage; ‘n_estimators’, the number of trees; ‘colsample_bytree’, subsample ratio of columns when constructing each tree; ‘colsample_bylevel’, subsample ratio of columns for each level; ‘scale_pos_weight’, balance of positive and negative weights. DNN hyperparameters: GeLU, Gaussian Error Linear Unit; dense dropout, fraction of the input units to drop.

## Data Availability

The data presented in this study are available on request from the corresponding author.

## Abbreviations

The following abbreviations are used in this manuscript:

AI	Artificial Intelligence
XAI	Explainable Artificial Intelligence
XGBoost	eXtreme Gradient Boosting
SHAP	Shapley Additive Explanations
AUC-ROC	Area under Curve-Receiver Operator Curve
CT	Computer Tomography
DS	Disease Score
ECOG	Eastern Cooperative Oncology Group
EOC	Epithelial ovarian cancer
FIGO	Federation International of Obstetrics and Gynaecology
IDS	Interval debulking surgery
PDS	Primary debulking surgery
CEA	Carcinoembryonic antigen
HE4	Human Epididymis 4
NHS	National Health System
ML	Machine Learning
NACT	Neoadjuvant chemotherapy
ACT	Adjuvant Chemotherapy
PPM	Patient Pathway Manager
MDT	Multidisciplinary team
BGCS	British Gynaecologic Cancer Society
CPEX	Cardiopulmonary exercise
ESGO	European Society Gynaecological Oncology
CCU	Critical care admission
SD	Standard deviation
CV	Cross validation
IMO	Intra-operative mapping of ovarian cancer
PCI	Peritoneal Cancer Index
NSQIP	National Surgical Quality Improvement Program
PS	Performance status
RD	Residual disease
R0	No residual—complete cytoreduction
SCS	Surgical complexity score
SJUH	St James’s University Hospital
